# Molecular Characterisation of Colour Formation in the Prawn *Fenneropenaeus merguiensis*

**DOI:** 10.1371/journal.pone.0056920

**Published:** 2013-02-18

**Authors:** Nicole G. Ertl, Abigail Elizur, Peter Brooks, Anna V. Kuballa, Trevor A. Anderson, Wayne R. Knibb

**Affiliations:** 1 University of the Sunshine Coast, Sippy Downs, Queensland, Australia; 2 Australian Seafood Cooperative Research Centre, South Australia, Australia; National Institute of Environmental and Health Sciences, United States of America

## Abstract

**Introduction:**

Body colouration in animals can have a range of functions, with predator protection an important aspect of colour in crustaceans. Colour determination is associated with the carotenoid astaxanthin, which is taken up through the diet and stabilised in the tissues by the protein crustacyanin. As a variety of genes are found to play a role in colour formation in other systems, a holistic approach was employed in this study to determine the factors involved in *Fenneropenaeus merguiensis* colouration.

**Results:**

Full length *F. merguiensis* crustacyanin subunit A and C sequences were isolated. Crustacyanin subunit A and C were found in the *F. merguiensis* transcriptomes of the muscle/cuticle tissue, hepatopancreas, eye stalk and nervous system, using 454 next generation sequencing technology. Custom microarray analysis of albino, light and dark *F. merguiensis* cuticle tissue showed genes encoding actin, sarcoplasmic calcium-binding protein and arginine kinase to be 4-fold or greater differentially expressed (*p*<0.05) and down-regulated in albinos when compared to light and dark samples. QPCR expression analysis of crustacyanin and total astaxanthin pigment extraction revealed significantly (*p*<0.05) lower crustacyanin subunit A and C gene transcript copy numbers and total astaxanthin levels in albinos than in the light and dark samples. Additionally, crustacyanin subunit A and C expression levels correlated positively with each other.

**Conclusions:**

This study identified gene products putatively involved in crustacean colouration, such as crustacyanin, sarcoplasmic calcium-binding protein and forms of actin, and investigated differences in gene expression and astaxanthin levels between albino, light and dark coloured prawns. These genes open a path to enhance our understanding of the biology and regulation of colour formation.

## Introduction

Nature's play with colours and different shades and patterns can have a variety of functions in animals, from thermoregulation, mate selection and communication to defence against predators [1], [2], [3], [4]. The latter appears to be the main role of body colouration in aquatic animals, such as prawns. For instance, some prawns protect themselves from predators by changing their body colour intensity to better adapt to their surroundings, while others use their transparent body combined with disruptive colouration to disguise their body outline from predators [1], [3], [5], [6]. Additionally, body colouration can also protect these aquatic animals from UV radiation, ensuring their continued health [7]. Aside from its function for the animal, colour in crustaceans also has commercial implications due to consumer perception and preference. This means, for example that darker coloured prawns are sold at a higher price per kilo than lighter ones [6], [8], making the darker prawns more valuable for the aquaculture industry. Of the various pigments existing in crustaceans, the fat soluble carotenoids appear to be the most important in the colouration of these animals, and are usually taken up with their diet or by a symbiotic relationship with organisms that produce these pigments [8], [9], [10], [11]. Astaxanthin, one of the carotenoids found in nature, appears to be the main pigment responsible for colour in crustaceans, including the prawn species *Fenneropenaeus spp*. [6], [8], [10], [12], [13], accounting for approximately 65% to 98% of all the carotenoids found in this species [8]. Crustaceans obtain this pigment from natural sources (e.g. green algae) or food supplements (in aquaculture production systems) and after ingestion of astaxanthin, the pigment is transported through the digestive system to the epidermis, where it is stored in subepidermal chromatophores [7], [8], [14]. Other than the epidermal layer, astaxanthin is also found in the exoskeleton, with the literature indicating that crustacean colouration is mainly due to the astaxanthin detected in these two locations [6], [11], [13]. Additionally, the quantity and distribution of astaxanthin also plays a role in the colour intensity of crustaceans [7], with crustaceans influencing their body colouration by 1) adjusting the amount of pigment in the chromatophores and exoskeleton, 2) changing the amount of chromatophores present in a tissue and 3) dispersing or concentrating the pigment inside the chromatophores [3], [6], [15].

Stability of the highly reactive astaxanthin pigment is provided by crustacyanin, a protein that binds to the pigment to form a carotenoprotein complex, which stabilizes the pigment as well as the protein's tertiary and quaternary structure [11], [16]. In the lobster *Homarus americanus*, binding of astaxanthin and crustacyanin occurs in the endo- and exocuticle, where β-crustacyanin is generated, after which the complex is moved into the epicuticle where α-crustacyanin forms [7]. The β-crustacyanin form is comprised of two protein subunits, bound to two astaxanthin molecules shared between the protein subunits, with α-crustacyanin being formed by eight β-crustacyanin heterodimeric molecules, irreversibly bound to each other and carrying a total of 16 astaxanthin molecules [9], [11], [17], [18], [19]. In the lobster *Homarus gammarus*, two types of protein subunits were detected, type 1 and type 2, also referred to as crustacyanin C and crustacyanin A, respectively. Type 1 includes protein subunits A1, C1 and C2, and type 2 is comprised of subunits A2 and A3, with each subunit being able to bind one astaxanthin molecule. Different combinations of type 1 with type 2 protein subunits then combine to form a β-crustacyanin heterodimer [9], [11], [12], [17], [18].

Once the non-covalent bond connecting the astaxanthin molecules with crustacyanin is cleaved by either cooking or dehydration, the carotenoproteins denature. This causes the relaxation of the hydrogen bonds holding the carotenoid, leading to the release of the pigment [17], [20], with the characteristic red colour of cooked crustaceans imparted by this released astaxanthin [21], [22]. Furthermore, it is argued that the strength of the colour depends on the amount of astaxanthin that had been bound to crustacyanin [21], [22].

In addition to astaxanthin and crustacyanin, a variety of other factors are likely to be involved in crustacean colouration. Liu et al. [23] analysed colouration in the tomato fruit and indicated that a suite of genes, not involved in the pigment synthesis pathway, appear to play a role in colouration by impacting positively on the amount of plastids and pigment produced, as well as by regulating the accumulation of the pigment lycopene. Billingham & Silvers [24] and Little [25] on the other hand focused on melanocytes, melanin and the genes involved in skin and hair colouration in a variety of animals and indicated that some of the genes they detected appeared to function in controlling 1) the amount and type of pigment synthesised, 2) the availability of products necessary for pigment formation, 3) the way in which the pigment granules were deposited, as well as their shape and size.

This study was carried out in order to enhance our understanding of the factors involved in colour formation in the prawn *Fenneropenaeus merguiensis*. Here, novel genes potentially associated with prawn colouration were identified using custom microarrays. Additionally, crustacyanin gene transcript expression levels and astaxanthin levels were determined in albino, light and dark coloured *F. merguiensis* prawns.

## Methods

### Animal husbandry

*F. merguiensis* samples used in this study were kindly provided by Seafarm and were reared in large aerated grow-out ponds. In an effort to ensure consistent environmental conditions across the different ponds, parameters such as feeding regimes and aeration were standardised across the ponds, and water quality monitored daily (e.g. pH, temperature and salinity). Dark and light prawns were randomly collected from one pond to minimize environmental variation. Albino prawns occurred only rarely and hence were collected from multiple ponds. All prawns were fed a starter feed (CP or Ridleys, Australia) until the animals had reached a weight of about 7 g, after which their diet was switched to a grower feed (CP or Ridleys, Australia) which contained 30 ppm/kg astaxanthin and was fed 4 times daily. Light and dark coloured prawns were randomly collected during the normal harvesting time and physiologically moult staged by testing the hardness of the carapace to obtain animals in intermoult, whereas the albino prawns were microscopically moult staged (three in late postmoult, two in intermoult and five in early to late premoult). Due to the rarity of the albino prawns all moult stages were included in the analysis.

### Crustacyanin subunit A and C gene sequence isolation

#### RNA extraction and cDNA synthesis

Total RNA was extracted from adult *F. merguiensis* epithelial and muscle tissue samples, using the RNeasy Plus Mini kit (Qiagen, Victoria, Australia), and cDNA synthesised from the isolated RNA. In order to obtain the full length sequence of both A and C crustacyanin subunits, 5' and 3' cDNA was synthesised from 460 ng and 1 µg of total RNA with the SMART^TM^ Race cDNA kit (Clontech, US) and the SuperScript^TM^ III First-Strand Synthesis SuperMix kit (Invitrogen Life Technologies, Victoria, Australia), respectively.

#### Primer development

Degenerate primers for crustacyanin subunit A and C were developed from conserved amino acid regions of four crustacean species (*Marsupenaeus japonicus*, GenBank: ACL37116; *Penaeus monodon*, GenBank: ACL37117 and *Litopenaeus vannamei*, GenBank: DQ858916 for subunit A; *M. japonicus*, GenBank: ACL37122; *Cherax quadricarinatus*, GenBank: ACL37121 and *P. monodon*, GenBank: ACL37123 for subunit C) and synthesised by GeneWorks (Hindmarsh, Australia). All primer sequences are presented in Table 1.

**Table 1:** Degenerative primers used for the isolation of crustacyanin subunit A and C.

Primer name	Sequence 5' → 3' *	Crustacyanin
A1F	TAY CAR CCN TAY AC	subunit A
A2F	GGN AAR ATH TAY CCN ACN AAN GAY TTY CC	subunit A
A2R	GGR AAR TCN TTN GTN GGR TAD ATY TTN CC	subunit A
A3R	AAN ACR AAN CCR AAY TC	subunit A
A4R	GTR TCR TAR TCN GTN TC	subunit A
C1F	CCN AAY CCN TTY GGN GAR CCN CA	subunit C
C1R	TGN GGY TCN CCR AAN GGR TTN GG	subunit C
C2R	GAY ACN GAY TAY GAR AA	subunit C

* **N** = A, C, T or G; **Y** = C or T; **R** = A or G; **H** = A, C or T; **D** = A, G or T.

#### Cloning of crustacyanin subunit A and C

Synthesised 5' and 3' cDNA were PCR amplified using the primers in Table 1.

PCR thermal cycling conditions were 1 min at 95°C, followed by 35 cycles of: 30 sec at 94°C, 30 sec at 39°C and 30 sec at 72°C, with the final extension step at 72°C for 10 min. The amplification reactions were carried out on 1 µl of template cDNA added to 200 nM each of the respective forward and reverse primer, 2.5 µl of 10×PCR reaction buffer, 2 mM of MgCl_2_, 200 µM of dNTPs, 1 U of Taq (reagents from Fisher Scientific, Australia) and RNase free water (G Biosciences®, St. Louis, USA) to obtain a total reaction volume of 25 ml. PCR products were purified with the QIAquick Gel Extraction kit (Qiagen) and ligated to the pGEM®-T Easy vector (Promega, USA), with the transformed cells grown and selected as outlined in the manufacturer's guidelines. Colony PCRs were carried out on white colonies, using the M13 Universal forward and reverse primers (GeneWorks) added to the PCR reagents and volumes described above to obtain a total reaction volume of 25 ml. Colony PCR thermo cycling conditions were: initial heat step at 95°C for 5 min, followed by 25 cycles of denaturation at 94°C for 30 sec, annealing at 48°C for 30 sec and extension at 72°C for 2 min, with the final extension at 72°C for 10 min. Nested PCRs were performed on the amplified colony PCR products to verify the amplification of the correct gene product, using primers C1F and C2R for the crustacyanin subunit C inserts and primers A4F and A3R for the crustacyanin subunit A inserts, with the PCR reaction volumes and cycling conditions as described for the cDNA PCR amplifications above. Gene sequences obtained from the 3' and the 5' RACE cloning were sequenced by commercial services (Macrogen, Korea and Australian Genome Research Facility [AGRF], Brisbane) and the sequences aligned with Sequencher v4.1 (Gene Codes, USA) to obtain the full length crustacyanin subunit A and subunit C gene sequences. Full length coding sequences for crustacyanin subunit A (GenBank: HM370278) and crustacyanin subunit C (GenBank: HM370279) were submitted to GenBank and their respective deduced protein sequences determined with InterProScan (http://www.ebi.ac.uk/Tools/InterProScan/).

### Transcriptome sequencing

#### Sample preparation

*F. merguiensis* samples (n = 75) of varying body colouration, gender and size (1–45 g) were randomly collected from eight different grow-out ponds, the cuticle and muscle tissue excised and stored in RNA Later ^TM^ (Ambion, Austin, TX).

Total RNA from the muscle and cuticle of the samples was extracted with the RNeasy Plus Mini kit (Qiagen), RNA quality and quantity assessed (1.2% ethidium bromide stained RNA gel and testing the A_260_/A_280_ ratio) and the RNA pooled into one composite sample, from which mRNA was isolated with the Oligotex mRNA kit (Qiagen) as per manufacturer's instructions. The mRNA was concentrated with the RNeasy Mini Elute ^TM^ Cleanup kit (Qiagen) and the prepared sample sent to AGRF for Roche 454 next generation sequencing.

#### Analysis of sequence reads

Raw sequence reads were processed with the CLC Genomics workbench software version 4.7 (CLCBio, Denmark) by removing the adaptor sequences from the single reads and *de novo* assembly of the transcriptome data into contigs, retaining the standard parameters set by the CLC software. Analysis and functional annotation of the transcriptome reads was performed, using the blastn function of the Blast2GO software (http://www.blast2go.org/start_blast2go) with the parameters set automatically by the software and the NCBI database.

#### Transcriptome data analysis of additional prawn tissues

Additional *F. merguiensis* tissue samples from the androgenic gland, hepatopancreas, stomach, nervous system, eyestalk and male and female gonads were prepared for next generation sequencing, applying the same extraction and analysis protocol as described above for the cuticle/muscle tissue. The obtained sequences were examined for the presence or absence of crustacyanin subunit A and C genes in these tissues.

### Colour analysis of albino, light and dark *F. merguiensis*

#### General sample preparation

Cuticle tissue was dissected from the first abdominal segment of dark (n = 20), light (n = 20) and albino (n = 10) adult *F. merguiensis* prawns and stored in RNAlater ^TM^ (Ambion). The prawn samples were then defrosted on ice until they reached a temperature of approximately 4°C, after which they were steamed at about 100°C for 4 min. Once the prawns were cooled on ice, they were photographed with a DMC-LZ3 Panasonic digital camera, set on flash and mounted 25 cm above the prawns. Photos were taken in a dark room, with the prawns placed on a white background. The prawn samples were then individually frozen at −20°C for 15 to 20 h.

#### Visual colour analysis

**Figure 1:**
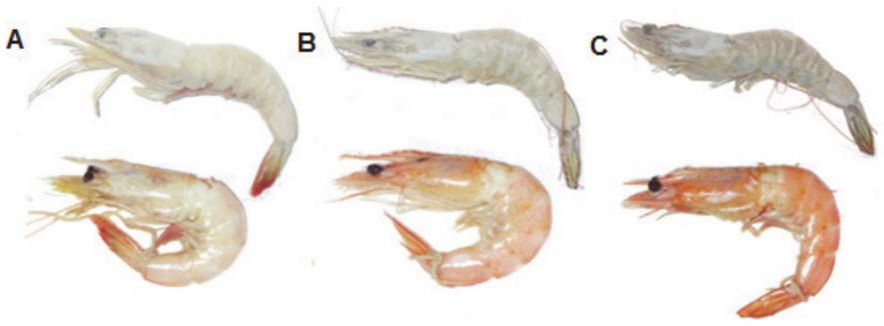
Example of cooked albino (a), light (b) and dark (c) *F. merguiensis* used in this study. Albino (a), light (b) and dark (c) prawns in the top row are uncooked, with a photo of the same animal after cooking directly below for comparison.

Colour groupings were determined by comparing the photographs (Figure 1) taken from the cooked prawns with the *P. monodon* colour chart (courtesy Seafarm, commercially available: Aqua-Marine Marketing, Australia), concentrating on the segment between the second abdominal segment and the telsons/uropods. Photographs were uploaded onto one single computer for colour ranking by University staff (n = 6).

#### Microarray design

A custom 4×44K microarray (Agilent, USA) was developed, using the analysed singletons and contigs from the cuticle/muscle tissue transcriptome, with the sequences chosen for the microarray being at least 100 bp long. PolyA tails, ribosomal sequences (e.g. 18S or 28S), as well as sequences that shared high homology (above 80%) across the full length of the sequence with other ribosomal RNA were removed from the dataset used for microarray development. As the orientation of the sequences obtained from the 454 transcriptome analysis was not known, the reverse complement sequences of the cleaned singletons and contigs were added to the microarray dataset along with the full length cloned crustacyanin subunit A and C sequences. Four probes (60 bp long) per sequence were designed by eArray and 212 control probes/replicates (printed in replicate 10 times on each array) were chosen from this probe list. The custom microarray data has been deposited in NCBI's Gene Expression Omnibus [26] under the GEO Series accession number GSE30346 (http://www.ncbi.nlm.nih.gov/geo/query/acc.cgi?acc=GSE30346).

#### Samples for microarray, quantitative PCR and pigment analysis

The mean and standard deviation values of the visual colour ranking results of the cooked light (n = 20) and dark prawns (n = 20) were statistically analysed (PASW Statistics 18) and the mean values sorted from the highest to the lowest number (excluding the 10 albino samples). Prawns with the 12 highest mean values (classified “dark” for the microarray experiment) and 12 lowest mean values (classified “light”) were chosen for hybridisation onto microarrays. For the samples classified “albino”, the 9 albinos with the visually palest perceived colouration were chosen. Random numbers were generated with Microsoft Office Excel 2007 (Microsoft Corporation, Australia) to randomly divide the samples into four groups each of their respective albino, light and dark colour. Each light and dark group was comprised of 3 prawns, whereas the 9 albino prawns were divided into three groups of two individuals and one group of three individuals. The same individual prawn samples were used for the microarray, qPCR and total astaxanthin analysis.

#### Sample preparation and hybridisation of microarrays

**Figure 2:**
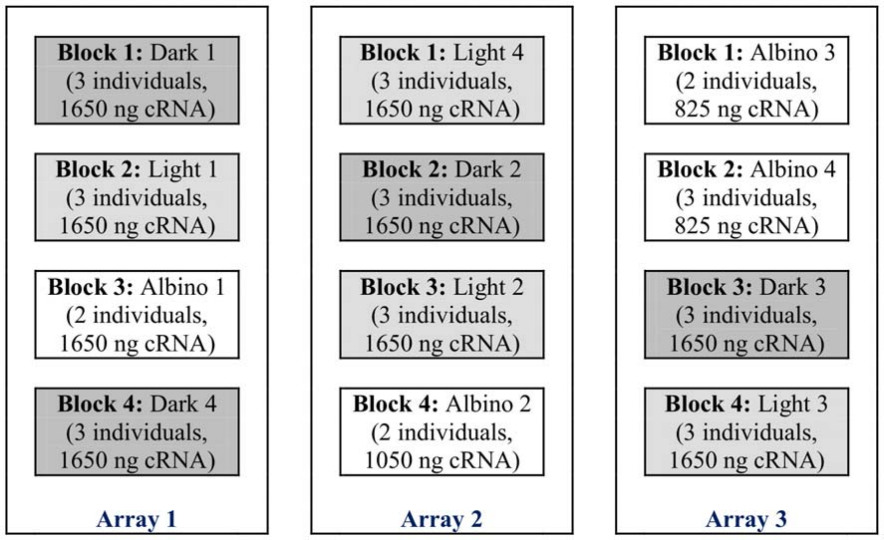
Hybridisation layout of the albino, light and dark groups across three 4×44K microarrays.

Total RNA from 20.0 mg of cuticle of the chosen dark (n = 12), light (n = 12) and albino (n = 9) *F. merguiensis* prawns was extracted with TRIzol® (Invitrogen Life Sciences). RNA integrity and purity was tested with gel electrophoresis (1.2% ethidium bromide stained RNA gel, Figure S1), NanoDrop2000 (A_260_/A_280_ ratio; Thermo Fisher Scientific) and a representation of the albino, light and dark samples were also tested with the 2100 Bioanalyzer (Agilent Technologies, USA), using the RNA 6000 Nano Chip kit (Agilent Technologies). Labelled complementary RNA (cRNA) was synthesised from 540 ng of total RNA, using the One-Colour Microarray-Based Gene Expression Analysis (Low Input Quick Amp Labeling) kit (Agilent Technologies) according to the manufacturer's instructions. For the cRNA synthesis, each individual of a group contributed equal amounts of total RNA, which was then pooled for each group. Labelled cRNA was purified with the Qiagen RNeasy kit (Qiagen) and its quantity and quality, as well as the dye incorporation determined with NanoDrop2000. Hybridisation of the sample groups was done as outlined in Figure 2. Lower amounts of labelled cRNA for Albino groups 2, 3 and 4 were due to low cRNA concentrations post labelling and purification. Hybridisation reactions and the wash steps were carried out as recommended in the One-Colour Microarray-Based Gene Expression Analysis (Low Input Quick Amp Labeling) protocol and the microarrays scanned with the GenePix 4000B Microarray scanner (Molecular Devices, California, USA). Scan parameters were 100% power, pixel size of 5 µm, lines to average: 1, focus position of 0 µm and channel green at 532 nm. The photomultiplier (PMT) gain of the scanner was set at 500 and then raised by 10 units every consecutive scan until 700 was reached. Each microarray scan was visualised with GenePix®Pro 6.0 (Molecular Devices, California, USA).

#### Microarray analysis of albino, light and dark prawns

For the analysis of the microarrays, scans with an approximate level of 1% supersaturation of spots were chosen. Scans were visualised with GenePix 6.0, and intensity values of foreground minus background were imported into GeneSpring GX 11.0 (Agilent Technologies). The arrays were then normalised and analysed with GeneSpring GX 11.0 to identify genes that were differentially expressed across the three colour groups. Normalisation was carried out using the GeneSpring GX 11.0 program with a threshold raw signal of 1.0, a percentile shift algorithm to 75th percentile, and no base line transformation. As lower amounts of total labelled RNA were used for the 4 albino sample groups, a K-means clustering analysis was performed on these samples to determine whether the albino data was normalised successfully. Unpaired t-tests were used to statistically analyse the normalised microarray data in GeneSpring GX 11.0. Because multiple single pair-wise hypotheses were tested, and this is known to inflate the experiment-wise probability of type I errors, we controlled for the expected false discovery rate (FDR) using the Benjamini and Hochberg procedure [27], with the significance level (α) for all statistical tests set at 0.05.

#### Sample preparation, primer design and validation for qPCR

cDNA was synthesised from 500 ng of total RNA from the 33 *F. merguiensis* samples and from 500 ng of pooled *F. merguiensis* cuticle tissue (reference sample) with the QuantiTect Reverse Transcription kit (Qiagen), including negative reverse transcriptions (-RT) for the reference sample and a representation (10%) of each colour group.

Gene specific primers were developed from the cloned full length coding sequences of crustacyanin subunit A (GenBank: HM370278) and subunit C (GenBank: HM370279) sequences of *F. merguiensis* with the Primer3Plus software (http://www.bioinformatics.nl/cgi-bin/primer3plus/primer3plus.cgi) and synthesised by GeneWorks.

Validation of the gene specific primers (Table 2) by qPCR and melt curve analysis was performed with a Rotor-Gene 6000 thermal cycler (Corbett Research, Australia). The qPCR reactions were carried out on 1 µl of reference cDNA template, added to 5 µl of 2×SensiMix, 0.4 µl of EvaGreen dye (from SensiMix^TM^ HRM kit, Quantace, USA), 200 nM of forward and reverse primer, and RNase-free water for a total reaction volume of 10 µl. Reactions were performed in duplicates, including no template controls (NTCs) and –RT, with the following cycling conditions: initial holding step at 95°C for 15 min, then 40 cycles of 1) 95°C for 15 sec, 2) 60°C for 15 sec and 3) 72°C for 30 sec, with the last step set to acquire to Green. HRM (high resolution melting curve analysis) conditions for the qPCR were set to rise by 0.1°C each cycle from 70°C to 95°C, with acquiring on the HRM channel. Quantification and melt curve analysis was performed, using the Rotor-Gene 6000 software, version 1.7 (Corbett Research, Australia).

**Table 2:** Primer pairs for qPCR analysis of dark, light and albino *F. merguiensis* individuals.

Primer name	Primer sequence (5' → 3')	Tm (°C)	Amplicon size (bp)	Crustacyanin	Source sequence	Efficiency
SubA2F	CAG GGC AAG ATC TAC CCC ACA	64.5	175	subunit A	HM370278	0.957
SubA2R	GGG AGA ACA CGA AGC CGA AC	64.3				
SubC1F	TGC TGG CAC ATG GTA CGA AA	63.6	205	subunit C	HM370279	0.939
SubC1R	TGC CTC GTA GTC CAC GGA AA	64				

Standard curves for crustacyanin subunit A and C were prepared by PCR amplification of 175 bp and 205 bp long crustacyanin subunit A and C segments, respectively, using reference cDNA and primer pairs SubA1F and SubA2R (for subunit A) and primer pairs SubC3F and SubC2R (for subunit C) (Table 2). PCR amplification reactions were set up as initially described for the gene isolation, with the exception of using an annealing temperature of 60°C. Amplified products were purified, using the QIAquick PCR purification kit (Qiagen) and quantified with the NanoDrop2000 spectrophotometer. Copy numbers for both segments were calculated with the copy number calculator of the URI Genomics & Sequencing Center (http://www.uri.edu/research/gsc/resources/cndna.html), and a 20 point serial dilution prepared for both crustacyanin subunit segments. Both standard curves (for subunit A and C) were run in triplicates, along with triplicate –RT and NTCs for both primer pairs outlined in Table 2, using the qPCR parameters described above.

From the dilution curves produced for both crustacyanin subunits, the reaction efficiency (E) was calculated by the Rotor-Gene 6000 software, version 1.7 with the equation E = [10(−1/M)] −1, where M stand for the slope of the curve.

#### Absolute qPCR on albino, light and dark prawn individuals

Absolute gene expression levels of crustacyanin subunit A and C were determined by qPCR analysis, with all individual albino, light and dark cDNA samples, -RT and NTCs for each primer pair (Table 2) analysed in duplicates, and a melt curve analysis executed after each run. In order to control for inter run variability and to determine expression levels, each qPCR run contained one point of the respective standard curve in triplicate. qPCR reaction volumes, cycling parameters and melt curve analysis were as outlined above.

#### Determination of total astaxanthin levels in albino, light and dark prawns

Extraction of total astaxanthin from the 33 cooked albino, light and dark *F. merguiensis* prawns was based on the protocol described in Tume et al. [6]. Although Tume et al. [6] determined that about 95% of the pigment found in their test species (*P. monodon*) was astaxanthin and astaxanthin esters, a small amount of other carotenoids such as lutein may be included in the extracted pigment. Therefore, the extracted astaxanthin was termed “total astaxanthin”. All preparations and extraction steps were executed at 4°C, with the extractions and following absorbance measurements protected from the light to limit its effect on the photosensitive pigment.

The fifth abdominal segment of each cooked prawn sample was chosen for total astaxanthin extraction as this segment was least likely to have lost pigment during the cooking process. The segment and the attached pleopods were cut and weighed, and the exoskeleton carefully separated from the cuticle tissue, with both, tissue and shell used in the extraction process. A mixture of 0.05% butylated hydroxyl toluene (BHT) dissolved in 100% acetone (BioLab, Victoria, Australia) was used as a solvent to extract the pigment from the shell and tissue. Two overnight extractions (20 h and 22 h) were carried out with 10 ml of fresh solvent used in each extraction and the total solvent volume adjusted to a volume of 20 ml after the second extraction step. Absorbance readings were conducted on a UV-1800 Shimadzu UV spectrophotometer. A full range wavelength scan (350 nm to 750 nm) was carried out that confirmed the wavelength of 477 nm indicated in Tume et al. [6] for the total astaxanthin absorbance measurements. Duplicate measurements were carried out on each sample, using the 477 nm wavelength, and the mean absorbance and amount of total astaxanthin in µg/g of wet weight calculated.

#### Statistical analysis

In order to determine whether significant differences in pigment quantity and crustacyanin subunit A and subunit C gene expression levels existed between the three different colour groups (albino, light and dark), the non-parametric Kruskal-Wallis test with Bonferroni's correction was used to lower the risk of type I error. This test was chosen as the data violated the assumptions of normality (tested with Levene's and Shapiro-Wilk test). Furthermore, non-parametric Kendall's tau correlation analysis was carried out to determine whether a relationship existed between the two crustacyanin subunits A and C, as well as between the level of gene expression and the amount of total astaxanthin extracted from the tissue. All statistical analysis was performed using PASW Statistics 18 (SPSS Inc., Illinois) and the results reported as mean ±1 standard error, unless otherwise noted. The significance level (α) for all statistical tests was set at 0.05.

## Results

In this study, RNA extracted from *F. merguiensis* prawns was used to isolate the crustacyanin subunit A and subunit C gene sequences, genes that have been implicated in colouration in other crustaceans. Furthermore, *F. merguiensis* individuals of different colour intensities were analysed for their levels of gene expression (crustacyanin and other genes) in their cuticle tissue (endocuticle and outer epithelium), utilising three molecular approaches: next generation sequencing, microarrays and qPCR. Additionally, to obtain a broader picture of the factors involved in body colouration of *F. merguiensis*, astaxanthin was also extracted from the above differently coloured prawns and assessed in relation to the gene expression level of crustacyanin.

### Crustacyanin subunit A and C gene sequence isolation

**Figure 3:**
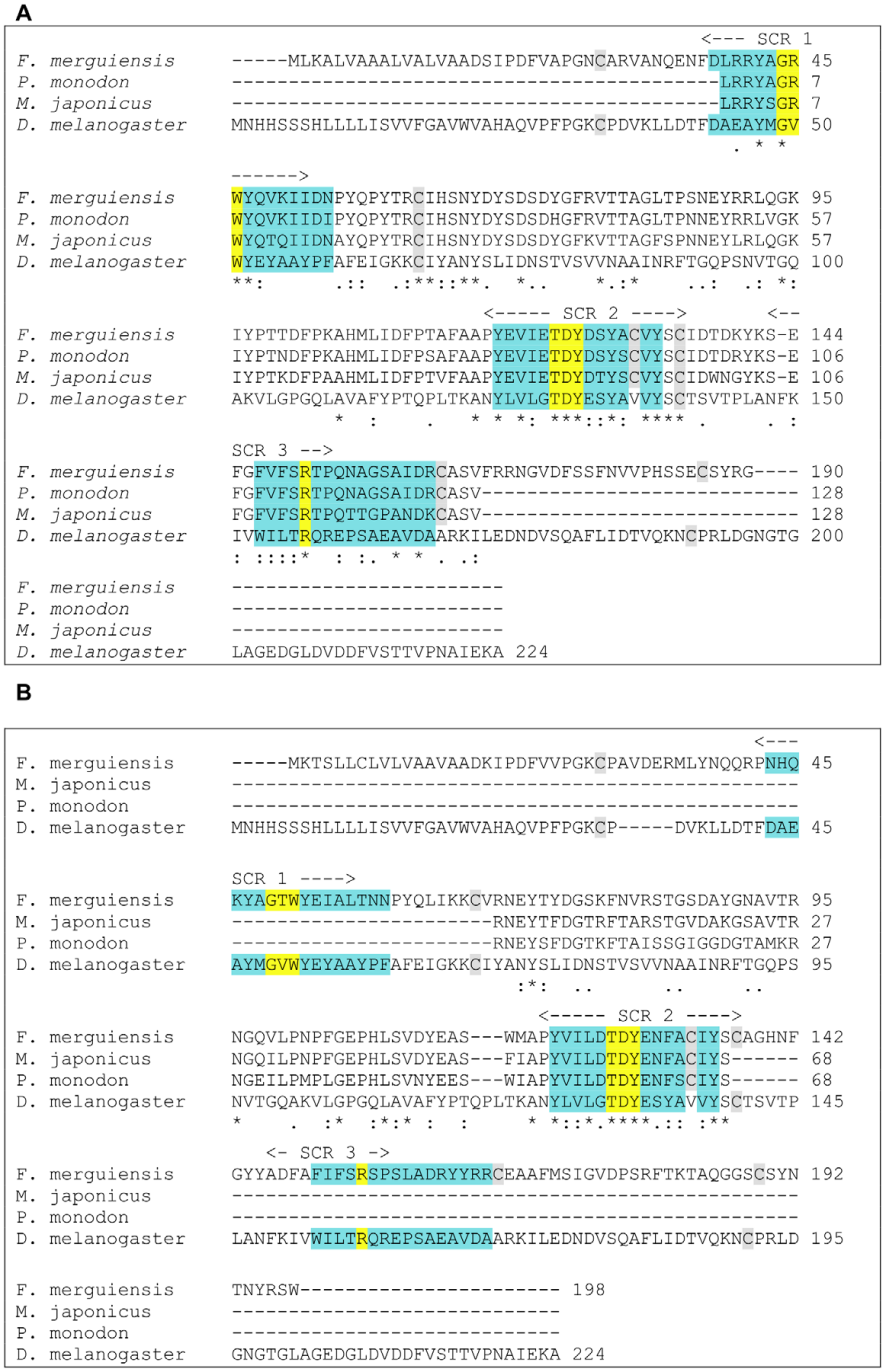
Protein alignment of lipocalins. *Drosophila melanogaster* neural lazarillo (GenBank: AAF51378) protein sequence aligned with **a)** deduced *F. merguiensis* crustacyanin subunit A protein sequence (GenBank: HM370278), partial *P. monodon* (GenBank: ACL37117) and *M. japonicus* (GenBank: ACL37116) crustacyanin subunit A protein sequences, and **b)** deduced *F. merguiensis* crustacyanin subunit C protein sequence (GenBank: HM370279), partial *P. monodon* (GenBank: ACL37123) and *M. japonicus* (GenBank: ACL37122) crustacyanin subunit C protein sequences. Blue highlighted segments correspond to motifs with the yellow inserts marking the conserved amino acids in these motifs. SCR stands for structurally conserved region, and grey highlights the six cysteine residues.

Full length consensus sequences of crustacyanin subunit A and C were isolated by cloning and submitted to GenBank (GenBank: HM370278 and HM370279 for subunit A and C, respectively). Analysis of the deduced protein sequences determined that both belonged to the large lipocalin family and were 35% homologous with each other. All of the three structurally conserved regions (SCRs) unique to the lipocalin family were found in the deduced protein sequences for crustacyanin subunit A and C, as well as the three motifs with their conserved amino acid sequence (GxW in motif 1, TDY in motif 2 and R in motif 3) [28], [29] and six cysteine residues (Figure 3).

To determine the locations of these SCRs and motifs, sequences from the study by Flower et al. [29] were used as a guideline. The deduced crustacyanin protein sequences were also compared with two partial crustacyanin subunit A and C protein sequences (GenBank: ACL37122 and ACL37123 for *Penaeus monodon* and *Marsupenaeus japonicus*, respectively) and neural lazarillo (*Drosophila melanogaster*, GenBank: AAF51378), another kernel lipocalin (Figure 3). Sequence homologies between the full length protein sequences of *F. merguiensis* crustacyanin and the partial crustacyanin sequences from *P. monodon* and *M. japonicus* were 94% and 83% for subunit A, and 68% and 84% for subunit C, respectively, while comparison with the lazarillo protein sequence showed homology mainly in the motifs and SCRs.

### Transcriptome sequencing

#### mRNA expressed in muscle/cuticle tissue

Roche 454 sequencing of the muscle/cuticle tissue resulted in 54928 sequence reads of between 30–540 bp segment lengths, from which 1455 contigs (between 45 bp and 4568 bp length) and 4535 singletons were derived. Analysis of the 5990 unique sequences with Blast2GO showed matches to sequences in the National Center for Biotechnology Information (NCBI) database for 3586 of these unique sequences, with functional annotations added to these sequences by Blast2GO. Closer examination of the functions of these sequences revealed that a large proportion had a role in cellular or metabolic processes, functioned in binding or had some catalytic or structural activity. Of the annotated sequences, only 0.04% were crustacyanin subunit A and C sequences (Figure S2) that showed high homology to the respective crustacyanin subunits isolated in this study.

#### Crustacyanin transcripts expressed in other tissues

Analysis of the transcriptome data obtained from six other *F. merguiensis* tissues revealed that crustacyanin subunit A and C gene transcripts were also expressed in the hepatopancreas, eye stalk and nervous system. No crustacyanin gene expression was detected in the stomach, androgenic gland and male and female gonads.

### Microarray analysis of albino, light and dark prawns

Custom microarrays were used to compare gene expression levels in the cuticle tissue of albino (n = 4), light (n = 4) and dark (n = 4) *F. merguiensis groups*. For the microarray analysis, signal intensity values were corrected for background signal and the data normalised in GeneSpring GX 11.0 (Agilent Technologies). Due to the differing quantities of labelled albino cRNA (825 ng, 1050 ng and 1650 ng) being hybridised to the custom microarrays, the validity of the normalisation of the albino samples was assessed with a cluster analysis (k = 20). A k = 20 was chosen to increase the likelihood that the patterns observed reflect true patterns. Visual analysis of the expression profile clusters with respect to cRNA concentration indicated a successful normalisation, with gene expression levels of the four albino groups not following the concentration gradient (Figure S3). Therefore, any variation observed in the different albino clusters was considered to be the response to genuine differences in the gene expression level of the different albino groups and was not attributed to the different amounts of labelled cRNA hybridised to the microarrays.

A principal component analysis (PCA) was performed on the normalised microarray data in order to identify overall patterns across the albino, light and dark colour categories in the microarray data. The clusters observed in the 3-dimensional scatter plot of the PCA scores revealed that gene expression of the albino samples was distinctly different to the light and dark samples and tightly clustered within itself. While the same pattern was seen for the dark samples, light samples appeared to be more broadly distributed, indicating that gene expression was potentially more varied in these samples (Figure S4). In order to visually confirm that position effects between microarrays (see hybridisation pattern in Figure 2) did not exist, an additional PCA ordination was plotted, identifying samples by their location on each hybridisation block on the microarrays. Visual inspection of the PCA scatter plot showed that no obvious clustering was discernible based on position of each sample on the microarray (Figure S5), indicating that the observed clustering based on colour was genuine.

Statistical analysis of the normalised microarray data, using unpaired t-tests with *p*-values corrected using the Benjamini and Hochberg procedure enabled the identification of gene products that were significantly differentially expressed between the three different colour groups. To determine similarities between the single pair-wise comparisons of albino and light and of albino and dark (Table 3), gene products that were statistically significantly (*p*<0.05) and 4-fold or greater differentially expressed in at least three out of four probes (of multiple probes reflecting a single gene), were determined in the first instance. For light and dark comparisons (Table 3), no statistically significant differential gene expression was found. In order to observe potential underlying biological trends across the colour groups, probes displaying a 4-fold or greater differential expression pattern (but not statistically significant) in at least three out of four probes (of multiple probes reflecting a single gene) were depicted for the pair-wise light and dark comparison. Analysis of the albino and light, and albino and dark single pair-wise comparisons (Table 3) showed that 28.6% (albino and light) and 30.6% (albino and dark) of the statistically significant differentially expressed probes were found to be unannotated, and 26.4% and 40.6% of the probes, respectively, represented forms of actin (e.g. beta-actin, actin 2) that were found to be significantly down-regulated in albinos. Gene probes coding for sarcoplasmic calcium-binding proteins and arginine kinase/allergen Pen m comprised the next highest group of significantly differentially expressed probes in the albino and light, and albino and dark comparisons and were also significantly down-regulated in albino samples. Furthermore, probes that coded for troponin I and to a lesser amount myosin light chains were also found in both comparisons (both significantly down-regulated in albinos). Other probes found to be statistically significant differentially expressed (down-regulated in albinos) were tropomyosin (14.8% in albino versus light) and elongation factor 1α and 2 (5.6% in albino versus dark).

**Table 3:** Microarray probes differentially expressed across albino, light and dark prawns.

	Number of Probes differentially expressed across the single pair-wise comparisons		
Gene ID	Albino and Light	Albino and Dark	Dark and Light
arginine kinase/allergen Pen m	16	13	0
Crustin	0	0	3
elongation factor 1 alpha	0	7	0
elongation factor 2	0	3	0
heat shock protein 70	0	0	4
myosin heavy chain	0	0	5
myosin light chain	3	3	0
possibly integrin-linked protein kinase 2	0	0	3
possibly male reproductive related protein	3	0	0
possibly myosin light chain	0	3	0
possibly solute carrier family 25 (phosphate carrier)	0	0	3
ribosomal RNA	0	0	71
sarcoplasmic calcium-binding protein	15	19	0
slow muscle myosin S1 heavy chain	0	0	6
slow tonic S2 tropomyosin	21	0	0
some type of actin	48	73	3
Tropomyosin	6	0	0
troponin I	18	4	0
Unannotated	52	55	54
**Total**	**182**	**180**	**152**

This table summarises the results of the single pair-wise comparisons for ease of readability, and shows probes that were 4-fold or greater and statistically significantly (*p*<0.05) differentially expressed according to the individual pair-wise comparisons of albino and light, albino and dark, and 4-fold or greater but not statistically significantly differentially expressed between light and dark *F. merguiensis*, in at least three out of four probes. The comparison between light and dark, although not significant, was included to show potential underlying biological trends in the data.

To determine intersections between the genes found in any of the three single pair-wise comparisons (albino versus light, albino versus dark, light versus dark), genes that were found to be statistically significantly (*p*<0.05) and 2-fold and above differentially expressed (not statistically significantly differentially expressed between light and dark) were displayed in a Venn-diagram. This diagram used the individual results from the three single pair-wise comparisons and graphically highlighted specific gene probe sequences that could be found significantly differentially expressed in, for example, only the albino versus light comparison and the albino versus dark comparison, but not in the light versus dark comparison. While pair-wise comparison between light and dark samples was not statistically significantly differentially expressed, the results of the comparison were included in this diagram to determine potential underlying trends in the data. From the genes found in the four overlap areas of the Venn-diagram, only genes that had at least two probes per gene sequence were examined further. The results showed that the majority of the probes shared between the three single pair-wise comparisons were again either unannotated, or were genes encoding forms of actin or sarcoplasmic calcium-binding protein (Table S1). Other probes identified in the overlap areas were from genes encoding QM protein, crustacyanin subunit A, cytochrome C oxidase, crustin, troponin I, tropomyosin, myosin heavy and light chain or arginine kinase/allergen Penm (Table S1). Crustacyanin subunit C probes were not observed in any overlapping comparisons.

Unannotated probes from 56 gene sequences were detected in every single pair-wise comparison in a relatively high percentage and analysed with Pfam (http://pfam.sanger.ac.uk/) and InterProScan for signal peptides and protein domains. Of these genes, 23 did not have a known domain or a signal peptide, and 12 did have a signal peptide but no domain. However, as most sequences derived from the transcriptome analysis were most likely partial sequences, the absence of a signal peptide in this analysis does not necessarily indicate that the full length sequence might not contain one. The 21 gene sequences that contained a functional domain are listed in Table 4. Domain functions of these 21 gene sequences varied, with the main function appearing to be either a binding, transport, regulatory or catalytic role.

**Table 4:** Protein domains and functions of 21 unannotated gene sequences differentially expressed in the microarray analysis.

Transcriptome ID	Signal peptide	Domain type*	Domain function
A86K5	Absent	cytochrome b	- electron carrier activity - oxidoreductase activity
ANCWJrev	Absent	Peptidase_M14	- metallocarboxypeptidase activity - zinc ion binding
AQEONrev	Present	MFS_general_subst_transpt	secondary membrane transporter
AY47U	Present	myosin head	- motor activity - ATP binding
B11UPrev	Absent	fibrillar collagen	extracellular matrix structural constituent
BXWCQrev	Absent	WAP-4-diS_core	peptidase inhibitor activity
C0173	Absent	Actin	protein binding
C95Y3rev	Present	ATPase_P-typ_ion-transptr	- ATP binding - ATPase activity, coupled to transmembrane movement of ions, phosphorylative mechanism
CFFRCrev	Absent	MFS_general_subst_transpt	secondary membrane transporter
CGC11rev	Absent	pyruvate kinase	- magnesium ion binding - pyruvate kinase activity - potassium ion binding
CIQIV	Present	Chitin_bind_4	structural constituent of cuticle
CJ2P2	Present	actin zf-C2H2	- protein binding - zinc ion binding
CMRY6rev	Present	zf-C2H2	zinc ion binding
Contig 1137rev	Absent	ATP-gua_Ptrans	- kinase activity - transferase activity, transferring phosphorus-containing groups
Contig 1413rev	Absent	RNA-binding S4 Blue (type 1) copper protein	- RNA binding - copper ion binding - electron carrier activity
Contig 227rev	Absent	zf-CCHC	- nucleic acid binding - zinc ion binding
Contig 905rev	Present	MFS_general_subst_transpt	secondary membrane transporter
CP026	Absent	ATP-gua_Ptrans	- kinase activity - transferase activity, transferring phosphorus-containing groups
DFP6W	Absent	Destabilase	lysozyme activity
EBSXErev	Present	Ser/Thr_prot_kinase-like	- protein serine/threonine kinase activity - ATP binding
EY7U2	Absent	AA_TRANSFER_CLASS_2 Lipocalin like	transferase activity binding

These unannotated gene sequences were found to be statistically significantly (*p*<0.05) and 2- or 4-fold and greater differentially expressed in the single pair-wise comparisons of albino versus light and albino versus dark groups, but not statistically significant between the light and dark groups. For ease of readability, protein domain results of the unannotated probes of each individual comparison were combined into one table.

* Abbreviations for domain types in Table 4: **ATP-gua_Ptrans**  =  ATP:guanido phosphotransferase, C-terminal catalytic domain; **zf-CCHC  = ** Zinc finger, CCHC-type; **WAP-4-diS_core**  =  Whey acidic protein, 4-disulphide core; **MFS_gen_subst_transpt**  =  Major facilitator superfamily, general substrate transporter; **Chitin_bind_4**  =  Insect cuticle protein; **AA_TRANSFER_CLASS_2**  =  Aminotransferase, class-II, pyridoxal-phosphate binding site; **Peptidase_M14**  =  Peptidase M14, caboxypeptidase D unit 2; **ATPase_P-typ_ion-transptr**  =  ATPase, P-type, K/Mg/Cd/Cu/Zn/Na/Ca/H-transporter; **zf-C2H2**  =  Zinc finger, C2H2-type; **Ser/Thr_prot_kinase-like**  =  Serine/threonine-protein kinase-like.

### Crustacyanin subunit A and C gene expression levels

**Figure 4:**
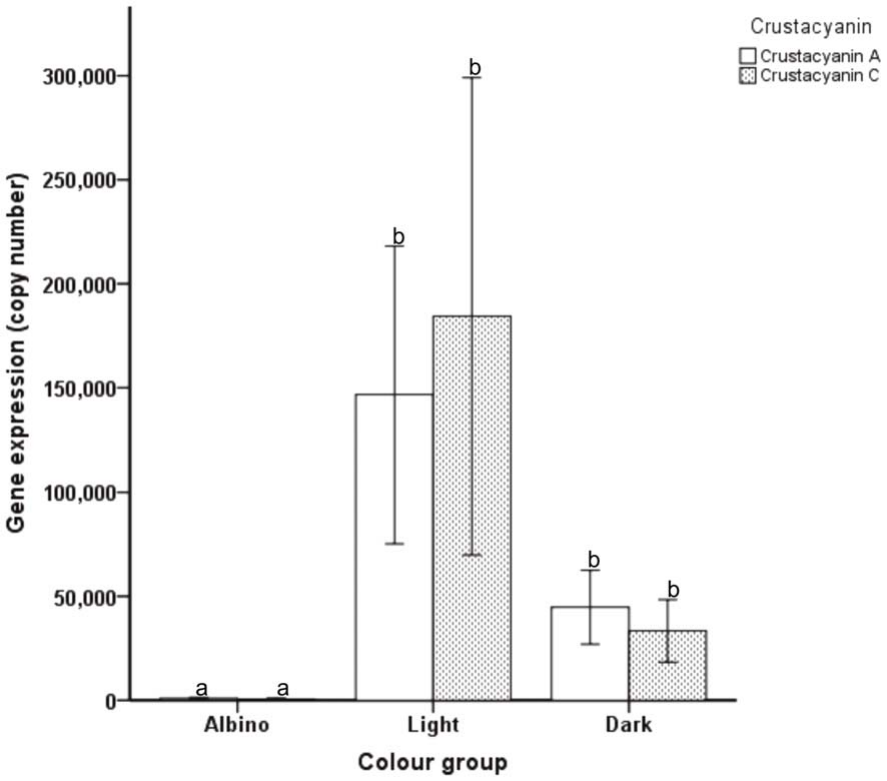
Crustacyanin subunit A and C gene copy numbers according to their colour groups (mean ± SE). Animals were separated into their respective colour groups (albino [n = 9], light [n = 12] and dark [n = 12]). Different superscripts (a, b) in the graphs represent significant differences (*p*<.05).

As crustacyanin is known to be associated with crustacean colour, qPCR analysis was carried out on the individual albino (n = 9), light (n = 12) and dark (n = 12) *F. merguiensis* prawns used for the microarray analysis, to verify the crustacyanin expression results observed in the custom microarrays. Analysis of the standard curve showed that crustacyanin gene copy numbers below 38.66 and 137.62 (crustacyanin subunit A and C, respectively) could not accurately be measured with qPCR. Therefore, values below this threshold were considered “below detectable limit” and assigned a value half of the respective lowest detectable limit for the statistical analysis. Crustacyanin subunit A and C gene transcript expression in albino prawns was minimal and significantly lower than the expression levels of both subunits in light and dark prawns, while expression levels between light and dark prawns were not significantly different (Figure 4). Comparison of crustacyanin subunit A and C gene copy numbers in each individual prawn revealed that crustacyanin subunit A was predominantly expressed at a higher level than subunit C (Table S2).

Correlation analysis of the qPCR data showed that the mean gene expression level of crustacyanin subunit A in albino, light and dark prawns was significantly positively correlated (*p*<0.01) with the mean copy number of crustacyanin subunit C in the same samples.

### Total astaxanthin analysis

**Figure 5:**
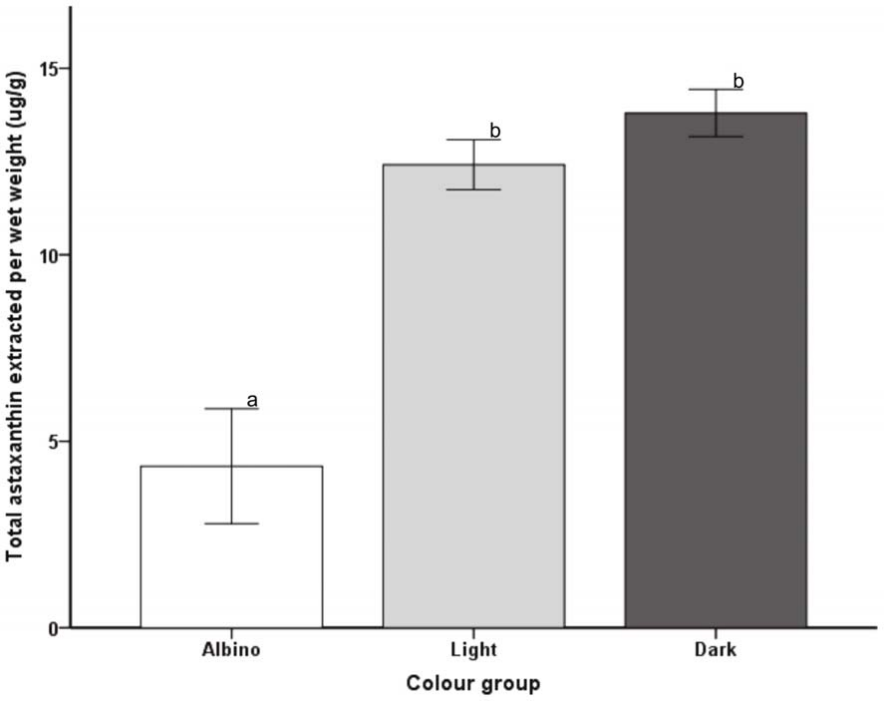
Total astaxanthin levels (mean ± SE) extracted from albino (n = 9), light (n = 12) and dark (n = 12) prawns. Pigment was extracted from the 5th abdominal segment of each of the 33 prawns. Different superscripts (a, b) in the graphs represent significant differences (*p*<0.05).

**Figure 6:**
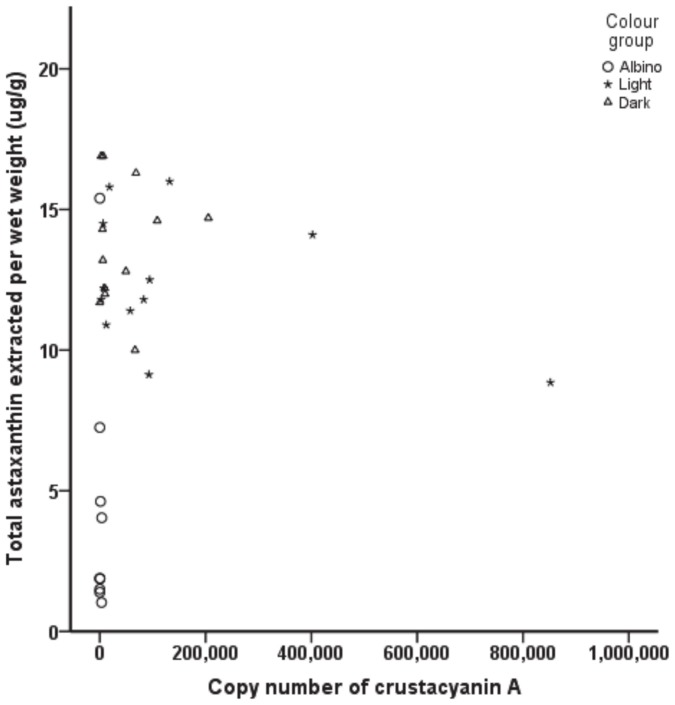
Scatter plot of crustacyanin subunit A and total astaxanthin levels. All colour groups were included into the scatter plot (0 = albino, 1 = light, 2 = dark), with crustacyanin subunit A and C showing the same pattern (subunit C not shown).

Chemical extraction of total astaxanthin from the exoskeleton and cuticle of the 5th abdominal segment was carried out on the 33 prawns used in the above gene expression analyses. Statistical analysis of the mean levels of total astaxanthin isolated from albino, light and dark prawns verified that albino prawns contained significantly lower amounts of total astaxanthin than light or dark prawns, with no significant difference in total astaxanthin observed between light and dark prawns (Figure 5). Correlation analysis of the mean total astaxanthin values of each of the three colour groups showed that there was no significant relationship (*p*>0.05) between the mean total astaxanthin values and the mean copy numbers of crustacyanin subunit A and C of each colour group. However, when crustacyanin subunit A and C, and astaxanthin were analysed irrespective of prawn colour, a common pattern was observed. A scatter plot (Figure 6) showed that an increase in crustacyanin gene copy number did not seem to reflect an increase in total astaxanthin levels, with light and dark prawns grouping together, while the albino samples clustered separately. The same pattern was observed for both crustacyanin subunits.

## Discussion

In this study, a combination of molecular and biochemical techniques, including qPCR, transcriptomics and custom microarrays were employed to improve our understanding of the factors involved in the colour variation observed between different individuals of *F. merguiensis*.

### Crustacyanin subunit A and C gene sequence isolation

Full length *F. merguiensis* crustacyanin subunit A and C gene sequences were successfully isolated. The coding nucleotide sequence isolated for crustacyanin subunit C was slightly longer (597 bp) than the sequence isolated for crustacyanin subunit A (573 bp). Analysis of the deduced protein sequences showed that both crustacyanin subunits contained the three SCRs and motifs with the conserved core amino acids (GxW, TDY and R) [30] typically found in proteins belonging to the kernel lipocalin family. Furthermore, the overall pattern of cysteine residues shared by proteins of the lipocalin family was also found in the *F. merguiensis* crustacyanin subunit A and C sequences, with the observed six cysteine residues having the potential to form three disulphide bridges. This is consistent with the literature which groups proteins that show one, two or all three SCRs and motifs into the lipocalin superfamily, with the kernel lipocalins encompassing proteins such as crustacyanin or retinol-binding protein that contain all three SCRs in their sequence [11], [31], [32]. In addition to the SCRs and motifs, conserved core amino acids of the three motifs can be found across the lipocalin group and are considered an important characteristic of this superfamily [11], [28], [29], [33]. Another characteristic of the lipocalin family is the presence of conserved cysteine residues (up to six residues) in the protein sequence [32], [33], which form disulphide bridges and have been found in the crustacyanin subunits isolated in this study (Figure 3) and in the subunits found in other crustacean species [11].

Alignment of the deduced *F. merguiensis* crustacyanin subunit A and C protein sequences with each other showed a similarity of 35% between the two sequences (Figure 3). This was consistent with other studies, which reported that protein sequence similarity in lipocalins is often not more than 30% [32]. Furthermore, Wang et al. [33] detected lipocalins that showed a similarity in protein sequence to other lipocalins (e.g. crustacyanin) of between 24% to 59%, and Cianci et al. [17] observed a 35% identity between the crustacyanin subunit A and C protein sequences isolated from a lobster species. Incidentally, Cianci et al. [17] also found that the crustacyanin subunit A protein sequence of their study was slightly shorter than the one for subunit C, as was observed for the *F. merguiensis* crustacyanin subunits in this study. These findings further confirm that the two isolated *F. merguiensis* crustacyanin subunits belong to the lipocalin superfamily, and more importantly, show the same characteristics of crustacyanin that have been observed in other studies.

### Transcriptome sequencing

Next generation sequencing was used in this study to assess the genes expressed in the muscle/cuticle tissue of *F. merguiensis* prawns. From this tissue, 54928 total sequence reads, with a read length of ∼30 to 540 bp were obtained through Roche 454 next generation sequencing, with the majority of the sequences having a length of approximately 250 to 450 bp. These results are quite typical of 454 next generation sequencing that has been reported to generate a large amount of sequence reads of roughly 300 to 400 bp length on average [34], [35].

The 1455 contigs and 4535 singletons generated from the 54928 total sequence reads were analysed with Blast2GO, and the results showed that around 40% of the unique sequences obtained were unannotated. This was expected, as crustacean sequences and functional information are limited compared to other model organisms. Examination of the overall distribution of functional annotations given to the 3586 unique muscle/cuticle transcriptome sequences by Blast2GO showed that the most predominant functional categories in this tissue were cellular and metabolic processes, as well as binding, catalytic and structural activity. This trend has similarly been observed in the transcriptome analysis of the greenhouse whitefly (*Trialeurodes vaporariorum*) [36] and the water flea (*Daphnia pulex*) [36]. Overall, this suggests that the transcriptome data obtained in the present study is an accurate representation of the genes expressed in the muscle/cuticle tissue of *F. merguiensis* prawns. Crustacyanin subunit A and C sequences comprised 0.04% of the 3586 annotated sequence reads from the next generation project and aligned strongly with the two sequences isolated through cloning. In addition to crustacyanin subunit A and C having been expressed in the muscle/cuticle tissue of *F. merguiensis*, both subunits were also detected in the transcriptome of the eye stalk, nervous system and hepatopancreas of this prawn species, with the transcript sequences bearing high homology to the two cloned sequences for subunit A and C. Complimentary to this study, Wade et al. [11] also isolated crustacyanin subunit A from the eye stalks, muscle and epithelial tissue of the lobster *Panulirus cygnus*.

While there did not appear to be any information in the literature regarding the functionality of crustacyanin subunit A and C in the eye stalk, nervous system or hepatopancreas, the lipocalin superfamily, to which crustacyanin belongs, is known to have a variety of functions. For instance, this family of proteins generally binds small, hydrophobic molecules, such as steroids, carotenoids or pheromones and also functions in cryptic coloration, immune regulation, cell growth, olfaction, cell homeostasis, development of the nervous system and binding to receptors on the surface of cells [28], [29], [31], [33], [37]. Whereas some of the lipocalins are believed to be very specific in the ligand they bind, most lipocalins can bind a wide range of ligands [38], therefore crustacyanin might not be limited to binding the carotenoid astaxanthin, but could potentially also be able to bind other molecules such as steroids, for example. For instance, apolipoprotein D, a lipocalin related to the insect bilin binding protein and crustacyanin subunit A1, is synthesised by cells in the central nervous system, where it is believed to function in nerve regeneration, signalling, metabolism and the transport of arachidonic acid [31], [39]. Other lipocalins expressed in the developing nervous system of *Drosophila melanogaster* and grasshoppers are Lazarillo and two Lazarillo-like lipocalins that aid in the development of axons and guide the direction of their growth [31], [32], [33], [38]. Considering the pleiotropic roles of lipocalins, it is possible that crustacyanin has a similar function to apolipoprotein D or Lazarillo in the nervous system of *F. merguiensis* prawns.

Crustacyanin detected in the transcriptome of the eye stalk of *F. merguiensis* could have a similar function as the lipocalins purpurin or lipocalin-type prostaglandin D synthase (L-PGDS). L-PGDS has been found in a variety of tissues, such as the brain, heart, testis and the retinal epithelial cells (pigmented) as well as iris and ciliary body epithelial cells (non-pigmented) [40], [41], and is the enzyme responsible for the production of the prostaglandin PGD_2_ in the eye which is believed to play an active role in retinal function. L-PGDS is also thought to function as a transporter for retinoids and other lipophilic molecules from the retinal pigment epithelium to the photoreceptor cells in the eye [40]. Similarly, purpurin binds and transports retinol; however, purpurin has also been observed to be involved in cell differentiation and adhesion, as well as the survival of the retinal epithelium and neurons. Purpurin was found to be expressed in the photoreceptor cells of the developing and regenerating fish retina, with an increase in protein expression observed between the second and fifth day of optic nerve regeneration and a higher purpurin protein expression level in the retina of fish during early embryogenesis compared to mature fish [42], [43], [44]. As lipocalins L-PGDS and purpurin appear to share a function as transporters, it could be possible that the crustacyanin subunits detected in the eye stalk tissue of *F. merguiensis* have a similar role.

Crustacyanin is likely to serve a similar purpose in the hepatopancreas, as in the prawn's cuticle tissue, for instance, by binding to the carotenoid astaxanthin. Astaxanthin is believed to have an additional role to crustacean pigmentation, by playing a part in crustacean and fish health due to the pigments' antioxidant properties [6], [21]. These antioxidant properties are thought to, for example, aid crustaceans and fish in the resistance to stress caused by hypoxia [45]. The hepatopancreas of decapods not only functions in food digestion, nutrient absorption, storage and metabolism, but also as an initial layer of immune defence [46], [47]. Jiang et al. [46] analysed the proteins expressed in the hepatopancreas of *Fenneropenaeus chinensis* prawns exposed to short-term hypoxia and the proteins that were observed to be up-regulated in their study included crustacyanin subunit C1, as well as carboxypetpidase B and chitinase. While the authors were unable to explain the importance of the finding that crustacyanin subunit C1 was up-regulated in the hepatopancreas of their experimental species [46], their findings linked into the results presented later by Pan et al. [45], who assessed the antioxidant capacity of fish fed with dietary carotenoids (astaxanthin and β-carotene) during a short-term hypoxia event and found that fish fed with a diet supplemented with carotenoids had a 56% lower superoxide dismutase level, as well as a decreased glutathione peroxidise and alanine transaminase activity than the control fish without the carotenoid supplements. These findings indicate that the carotenoids fed to the fish had an increased antioxidant capacity, which potentially led to a higher tolerance to hypoxic stress events in these animals. Together with the above study by Jiang et al. [46], this suggests that the crustacyanin expressed in the hepatopancreas could serve as astaxanthin collector or transporter of astaxanthin and β-carotene to the tissues most affected by a hypoxic stress event, thus contributing to the overall wellbeing of the prawn.

### Microarray analysis

While the next generation transcriptome data allowed us to gain an understanding and overview of the genes found in the *F. merguiensis* muscle/cuticle tissue, it was also used to create a custom microarray to determine genes differentially expressed across the cuticle tissue of light, dark and albino coloured *F. merguiensis* prawns. By employing custom microarrays, the vast transcriptome dataset obtained in the present study was scanned for potentially novel genes involved in the colouration process of *F. merguiensis* prawns.

Normalised microarray data were analysed and gene probes significantly differentially expressed between the three colour morphsidentified. Analysis was restricted to probes that showed a 2- or 4-fold and greater differential expression in the cuticle tissue in individual pair-wise comparisons between albino and light, albino and dark, and light and dark groups, while probes that were significantly different, but less than 2-fold differentially expressed between the three colour groups were excluded. This cut-off was chosen to increase the likelihood that the identified gene probes had a genuine biological role in the animals of one colour group, compared to those from another colour group. Furthermore, as hybridisation efficiency can vary between individual microarrays, and as varying amounts of labelled cRNA were used for the albino samples (850 ng for two groups, 1050 ng and 1650 ng for one group each), emphasis was put on normalisation of the data. In addition, while albino samples of different moult stages (late postmoult, intermoult and early to late premoult) were used in this study, moult stage did not appear to have had any confounding effects. For example, genes such as Uca ecdysteroid receptor (UpEcR) and retinoid-X-receptor (UpRXR) gene homologs found to be differentially expressed in the hypodermis of the fiddler crab *Uca pugilator* across the C_4_ through to D_1-4_ moult stages [48] were not found in this study. Moreover, gene products previously found to be differentially expressed across the crustacean moult cycle, such as mannose-binding protein, C-type lectin receptor, trypsin-like and chymotrypsin-like, carcinin-like, clotting protein precursor-like, hemocyanin [49] and cuticle proteins containing the PfamB_109992 and CBM14 domain [50], were not found to be differentially expressed in this study. While the chitin_bind_4 domain (associated with cuticle proteins) [50] was found significantly differentially expressed between albino and light samples, it was not found differentially expressed in any of the other comparisons. More importantly, Kuballa et al. [50] noted that the chitin_bind_4 domain was up-regulated in intermoult when compared to early pre-moult. As light and dark samples of this study were in intermoult, it is therefore unlikely that the expression of chitin_bind_4 in our study was linked to moult cycle.

Examination of the overall distribution of the albino, light and dark colour categories with a PCA scatter plot (Figure S4) showed albino and dark samples to be tightly clustered within each group and distinctly different to each other, while the light samples appeared to have a broader distribution. This observation was consistent with the qPCR results of the present study where gene transcript expression levels of crustacyanin subunit A and subunit C in the albino samples were significantly different from the light and dark samples. Furthermore, correlating total astaxanthin amounts with crustacyanin gene expression levels irrespective of colour confirmed the pattern seen in the PCA scatter plot, with albino samples distinctly separate from light and dark samples (Figure 6). This indicates that while low levels of pigment and gene copy numbers appear to relate to the absence of colour (albino), high levels of pigment and gene expression did not seem to correlate with colour intensity (light and dark).

Analysis of the single pair-wise comparisons between albino and light, albino and dark, and light and dark showed an overall similar sequence distribution of differentially expressed genes (not statistically significant between light and dark, but added to identify underlying data trends across colours), with the highest number of 4-fold and greater differentially expressed probes found to be unannotated (28.6%–35.5%) or coding for forms of actin (2%–40.6%). As the Blast2GO analysis showed highly significant matches to a variety of actin forms (e.g. beta-actin, actin 2) for an individual *F. merguiensis* actin gene sequence, the term “forms of actin” was given to these gene products.

Down regulation of gene probes coding for forms of actin, sarcoplasmic calcium-binding protein, arginine kinase/allergen Pen m and troponin I was observed in albino prawns when compared to either light or dark prawns and appeared to play an important role in all three colour groups. This finding was confirmed, when the albino and light, albino and dark, and light and dark single pair-wise comparison results were combined into a Venn-diagram (Table S1) and were found to be 2-fold or greater differentially expressed across more than one colour group comparison.

As in this study, Mykles et al. [51] detected actin in the epithelium, and membranous and endocuticular layer of crab and lobster exoskeletons. The authors suggested that the epithelium of these crustaceans secreted actin, together with tubulin to enable the incorporation of these proteins into the extracellular matrix, where the proteins could function as stabilizers or organizers of the matrix. Other studies, however, suggest an additional function of these two proteins. Fingerman et al. [52], for instance, proposed that microtubules and microfilaments found in the fiddler crab *Uca pugilator* could play a role in the movement of pigment granules in the chromatophores of the animal. In their study, the authors used colchicines and cytochalasin B to disrupt the microtubules and microfilaments, and observed inhibition of pigment concentration and dispersion in ovarian erythrophores of the fiddler crab. Further studies appeared to have resulted in similar findings, as, for example, Beckerle & Porter [53] analysed granule movement in the erythrophores of the squirrelfish *Holocentrus ascensionis*, which the authors ascribed mainly to microtubules. However, there also appeared to be microtubule-independent movement that was believed to be due to actin microfilaments. Tuma & Gelfand [54] stated in their review of pigment granule movement in melanophores that both, microtubules and actin microfilaments played an important role in aggregation and dispersion of the pigment granules. Furthermore, they noted that the dispersion of the pigment granules and maintenance of this dispersed state required actin microfilaments. Research on pigment movement in retinal pigment epithelial cells coincides with the results obtained in chromatophores, as a study by Burnside et al. [55] suggested that dispersion of pigment granules in the retinal pigment epithelial cells was dependent on actin for the translocation of the pigment. Also, research carried out by McNamara & Ribeiro [56] indicated a potential role for actin in the slower phase of pigment aggregation.

With other research indicating that actin was necessary for pigment dispersion and maintaining this state in the animal [54], it is possible that the 2-fold and greater change in expression levels observed in gene probes coding for forms of actin in *F. merguiensis* prawns of this study reflected the colour, as well as the state of pigment dispersion or aggregation in these animals, as gene probes encoding actin were found to be down-regulated in albinos when compared to light or dark prawns. While expression levels of tubulin did not appear to have changed in these animals, it is possible that the amount of microtubules in the epithelium and exoskeleton of the *F. merguiensis* prawns did not change significantly, or that tubulin levels are regulated at the post transcriptional level. The findings of the involvement of microtubules and actin microfilaments in the movement of pigment could also explain the light and dark adaptation Tume et al. [6] observed in their study on the black tiger prawn *Penaeus monodon*, where the pigment was found to be aggregated in lighter environments, resulting in a lighter prawn than the prawns found in darker environments, where the pigment was shown to be dispersed.

Gene probes coding for troponin I, tropomyosin, sarcoplasmic calcium-binding protein, QM protein, arginie kinase and to a smaller extent, for myosin light and heavy chain also showed a 2-fold or greater change in expression level across the three colour groups in the microarray analysis of this study. The function of these proteins could potentially be linked to the action of actin in the cuticle tissue of *F. merguiensis* prawns of different body colouration. For example, in addition to the microtubules and microfilaments, different motors are thought to be important for the movement of granules in melanophores. Research has directed its focus so far on dynein and kinesin II as motors for pigment aggregation and dispersion, respectively. Also, myosin V is believed to be the motor that enables the transport of pigment across actin microfilaments, functioning in pigment dispersion [54], [56], [57]. Although neither dynein nor kinesin II were found to be 2-fold or greater differentially expressed across the three colour categories, small numbers of probes from myosin light and heavy chain genes were detected to be 2-fold and greater differentially expressed across the albino, light and dark prawns. As myosin V belongs to the myosin superfamily [58], [59], the 60 bp long probes of the microarray could have potentially shared sequence homology with other members of the superfamily that have already been annotated or are novel members of the family.

Genes encoding arginine kinase were also determined to be statistically significantly down-regulated in albinos when compared to light and dark prawns. Arginine kinase belongs to the phosphagen kinase family [60], [61] and functions as an enzyme, using arginine phosphate as a substrate to produce ATP from ADP. The produced ATP has a buffering effect by allowing maintenance of cell function over a short period of time when energy demand is higher than what oxidative phosphorylation can provide [62], [63], [64], [65]. As energy in form of ATP is needed for the transport of pigment (dispersion and aggregation), and potentially to maintain the dispersed state of the pigment granules, arginine kinase could play a role in temporarily providing the ATP necessary for this function.

Genes coding for QM protein, also found to be 2-fold and greater differentially expressed between the three colour categories, is thought to play a role in the immune system of invertebrates, but could also function in the actin cytoskeleton, as deletion of GRC5/QSR1, a homolog of the QM protein, resulted in an abnormal actin cytoskeleton [66]. Probes encoding troponin I and tropomyosin, two proteins directly associated with actin, were also identified to be 2-fold and greater differentially expressed across the albino, light and dark prawns. Both proteins are known to bind to actin filaments, where they are two of four proteins that regulate actin-myosin interactions, with the type of interaction dependent on the presence or absence of calcium [67], [68]. Genes encoding sarcoplasmic calcium-binding protein detected in the microarray analysis are also linked to actin and potentially to pigment motility, as this protein functions as a calcium carrier, moving the protein to where it is needed, mainly to stimulate contraction by permitting actin-myosin interaction [69]. Although speculative, the observed statistically significant and 2-fold or greater down regulation of sarcoplasmic calcium-binding protein expression in the albino prawns compared to the light and dark prawns could indicate that there were differences in calcium levels across the three colour categories that make it necessary to have increased or decreased amounts of this calcium carrier. This finding could be linked to reports in the literature that indicated a potential calcium involvement in the movement of pigment. For instance, Tuma & Gelfand [54] stated that increased intracellular calcium levels caused pigment aggregation, a pigment movement that was observed to be inhibited when no extracellular calcium was present. This was confirmed by Kotz & McNiven [57] that observed that an increase in calcium levels triggered the aggregation of pigment in erythrophores and an inhibition of aggregation when extracellular calcium was absent. Furthermore, the authors indicated that pigment dispersion relied on a decrease in calcium levels as well as an increase in cAMP levels. Considering the link between calcium levels and pigment aggregation, as well as dispersion, differential expression of the sarcoplasmic calcium-binding protein in the three different colour categories of the *F. merguiensis* prawns suggested that the movement of pigment in the chromatophore could function in a similar way as was observed for retinal pigment epithelial cells, melanophores and erythrophores.

Two elongation factors, elongation factor 1α and elongation factor 2 were other interesting genes found to be significantly down-regulated in the albino samples compared to the dark samples. While elongation factor 2 is specific in its function of regulating the elongation step in protein synthesis [70], elongation factor 1α appears to have a variety of functions. For instance, elongation factor 1α was indicated to have a role in cell proliferation along with zinc finger proteins, which were also found to be differentially expressed between the three *F. merguiensis* colour categories (Table 4). Elongation factor 1α has also been shown to have an actin bundling, as well as a microtubule severing ability; however, the signals or mechanisms that trigger this switch between bundling and severing action have not been established [71]. Whether or not the different elongation factors, particularly elongation factor 1α, have any function in prawn colouration remains to be determined; however, as actin microfilaments and microtubules have been shown to facilitate pigment transport, it is possible that elongation factor 1α could be directly or indirectly associated with pigment transport.

While a variety of gene products were found to be differentially expressed between albino, light and dark prawns, crustacyanin subunit A was only detected in 2.9% of the probes that were 2-fold or greater and statistically significantly down-regulated in the albino prawns compared to the light and dark prawns. These findings correlate with the results of the qPCR experiment of this study, where significantly lower crustacyanin subunit A gene copy numbers were found for the albino samples than for the light or dark prawns (Figure 4). Furthermore, individual gene expression levels of crustacyanin subunit A were predominantly higher than gene expression levels for subunit C, which has also been observed in the microarray analysis, were crustacyanin subunit A but not subunit C was detected in the 2-fold and greater differentially expressed probe list. Considering that crustacyanin subunit A and C are known to be important for colouration in crustaceans, it was interesting to observe that there was a 2-fold or lower change in expression level of the crustacyanin subunit A and C genes between the three *F. merguiensis* colour categories, compared to gene products such as the various forms of actin, troponin I or arginine kinase. These differences in expression levels could be caused by one of two factors. Either, only slight changes in the expression levels of crustacyanin subunit A and C maybe biologically significant and could cause a change in prawn colouration. Alternatively, crustacyanin subunit A and C genes might only be responsible for a small change in prawn colour, with other factors having a stronger influence on prawn colouration. The latter is supported by a study carried out by Wade et al. [72] that showed a difference in free astaxanthin levels, but not in crustacyanin gene expression levels, when prawns of different colour intensity were compared. In this study colour change was induced in *P. monodon* by exposure to either a light or a dark background and a consistent difference in colour intensity was detected; however, regulation of this adaptive colour response was not observed at the gene expression level.

### qPCR colour analysis and total astaxanthin determination

Crustacyanin subunit A and C gene transcript expression levels in the cuticle tissue of the examined 33 *F. merguiensis* prawns were determined with qPCR, using gene specific primers for both subunits that were designed from the cloned sequences of crustacyanin subunit A and C. Results of the qPCR experiment revealed that both, crustacyanin subunit A and C gene expression levels in albinos were significantly lower than in light or dark prawns, as was expected (Figure 4).

Correlation analysis of the crustacyanin subunit A and C gene transcript expression levels of the albino, light and dark prawns revealed a significantly positive relationship between the expression levels of the two subunits across the three different body colourations. This was expected, as the literature indicated that both subunits, crustacyanin subunit C (type 1) and crustacyanin subunit A (type 2) are needed to form a β-crustacyanin heterodimer, with both subunits sharing two astaxanthin molecules. Eight of these β-crustacyanin heterodimer would then bind together to form α-crustacyanin [9], [11], [17], [19]. A study that quantitatively analysed the expression of a gene similar to crustacyanin was carried out in white and red western rock lobsters (*Panulirus cygnus*) and found that there was no difference in gene expression between the two colour groups [73]. This suggests a potentially different physiological mechanism associated with the migratory phase of the juvenile western rock lobster (red to pale pink [termed white phase] colour change), when compared to the *F. merguiensis* prawns of different body colouration analysed in this study.

In addition to the determination of crustacyanin subunit A and C gene transcript expression levels in albino, light and dark prawns, levels of total astaxanthin were also assessed in the same 33 *F. merguiensis* prawns. Results of the total astaxanthin extraction and spectrophotometric analysis showed significantly lower amounts in albinos compared to light or dark prawns, with no significant difference in total astaxanthin levels found between light and dark prawns. Similar to the findings of this study, Wade et al. [13] correlated the total amount of carotenoids found in the western rock lobster shell and epithelium with the colour of the lobster shell, with lower levels of carotenoid found in the white lobster and higher levels in the red lobster. Tume et al. [6] on the other hand, determined that the quantity of astaxanthin extracted from *Penaeus monodon* prawns could not be closely associated with the visually observed colour of these prawns. Tume et al. [6] however, used pre-cooked commercial prawns and did not include albino prawns, which therefore make it difficult to compare directly to the results observed here.

## Conclusions

Analysis of albino, light and dark samples in this study gave an insight into potentially important factors contributing to the differences in colour intensities seen in *F. merguiensis* prawns. Gene expression analysis using qPCR and total astaxanthin extraction showed a significant difference of crustacyanin subunit A and C gene transcript expression and total astaxanthin levels in the albino compared to the light and dark samples. Additionally, microarray analysis identified gene products such as sarcoplasmic calcium-binding protein, arginine kinase and various forms of actin to be gene products that were differentially expressed and therefore appear to play a role in colour formation in *F. merguiensis* prawns and warrant further investigation. Furthermore, closer examination of the correlation between total astaxanthin and crustacyanin subunit A and C indicated that a decrease in gene and pigment levels seems to be associated with an absence of colour (albino) but not colour intensity (light and dark). Moreover, additional studies concerning genes identified in the microarray analysis will contribute to advances in the knowledge of the biology and regulation of colouration in crustaceans.

## Supporting Information

Figure S1 **RNA gel picture of the albino, light and dark prawns analysed in this study.** RNA gel visualisation of total RNA extracted from the cuticle tissue of the examined albino (ID# 173–177 and 180–183), light (ID# 1, 3, 6, 7, 14, 15, 19, 20, 22, 24, 25 and 91) and dark (ID# 11, 30, 86-88, 90, 93, 95, 97, 101, 104 and 109) *F. merguiensis* prawns.
(PDF)

Figure S2 **GO annotations for 3586 annotated** ***F. merguiensis*** **muscle/cuticle genes.** Pie charts of a) molecular function (level 2), b) cellular components (level 3) and c) biological processes (level 2) of the annotated genes found to be expressed in the muscle/cuticle tissue. Blast2GO software was used for gene annotation.
(PDF)

Figure S3 **K-means (k = 20) cluster analysis performed across the albino groups.** Vertical axis depicts normalised intensity values, horizontal axis shows the three different cRNA amounts (2×825 ng, 1050 ng and 1650 ng) used in the hybridisation reaction of the four albino sample groups, with the lowest concentration on the left and the highest on the right. Three of the albino groups were each comprised of cRNA from two pooled individuals, while the fourth albino group had three individuals contributing to the total cRNA amount (825 ng) for the hybridisation reaction.
(PDF)

Figure S4 **Principal component analysis (PCA) on albino, light and dark groups.** The PCA plot shows the overall pattern of distribution of the four albino (red circles), four dark (blue circles) and light (brown circles) prawn colour groups. Component 1 is shown by the x-axis (44%), component 2 by the y-axis (22%) and component 3 by the z-axis (19%). Percentages in brackets are the variance explained by each axis.
(PDF)

Figure S5 **Principal component analysis (PCA) based on position of each block on the microarrays.** The PCA plot shows the overall pattern of distribution of each of the hybridisation blocks on each of the three microarrays. PCA ordination was plotted with GeneSpring GX 12.5 (Agilent Technologies), depicting the relationships among samples on the basis of their respective locations on the microarrays: Block 1 (red circles, n = 3), Block 2 (blue circles, n = 3), Block 3 (brown circles, n = 3), and Block 4 (grey circles, n = 3). Component 1 is shown by the x-axis (explains 34% of the variation), component 2 by the y-axis (explains 12% of the variation) and component 3 by the z-axis (explains 10% of the variation).
(PDF)

Table S1 **Mutual expression profile of microarray probes differentially expressed across albino, light and dark prawns.** Table shows probes that were found in the overlap areas of a Venn-diagram that graphically highlighted specific gene probes found in more than one of the single pair-wise comparisons (albino versus light, albino versus dark and light versus dark). The probes were found to be 2-fold or greater and statistically significantly (*p*<0.05) differentially expressed between albino and light and albino and dark *F. merguiensis* in at least two out of four probes. Results of the single pair-wise comparison of light versus dark, although not statistically significant, were included into the Venn-diagram and successive table to examine potential underlying biological trends.
(PDF)

Table S2 **Individual crustacyanin subunit A and C copy numbers of the albino light and dark prawns.** Mean individual copy numbers for each *F. merguiensis* prawn were determined with absolute qPCR, with each sample run in duplicate.
(PDF)
