# Supplementary figures and images for: Molecular Characterisation of Colour Formation in the Prawn Fenneropenaeus merguiensis
Source: PLoS One. 2013 Feb 18;8(2):e56920. doi: 10.1371/journal.pone.0056920 (PMC3575496; doi:10.1371/journal.pone.0056920)

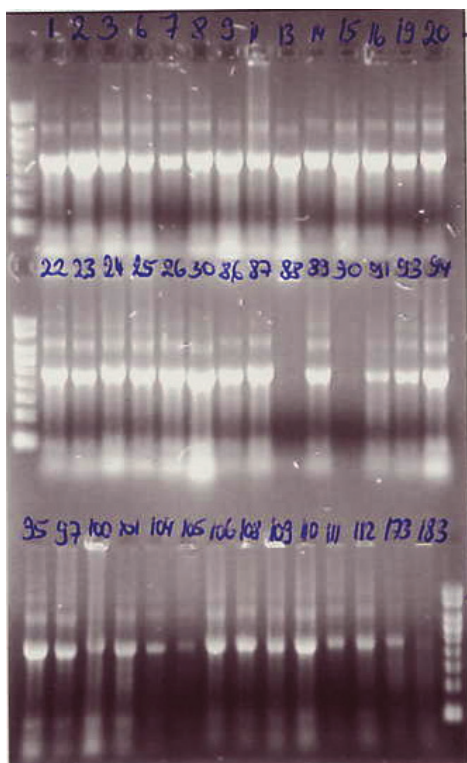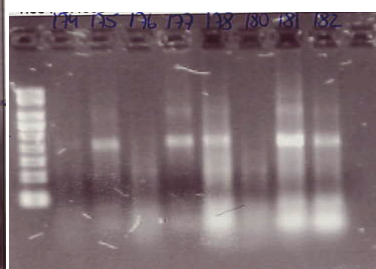

Supplement: Figure S1 — RNA gel picture of the albino, light and dark prawns analysed in this study. RNA gel visualisation of total RNA extracted from the cuticle tissue of the examined albino (ID# 173–177 and 180–183), light (ID# 1, 3, 6, 7, 14, 15, 19, 20, 22, 24, 25 and 91) and dark (ID# 11, 30, 86-88, 90, 93, 95, 97, 101, 104 and 109) F. merguiensis prawns. (PDF) [file pone.0056920.s001.pdf]

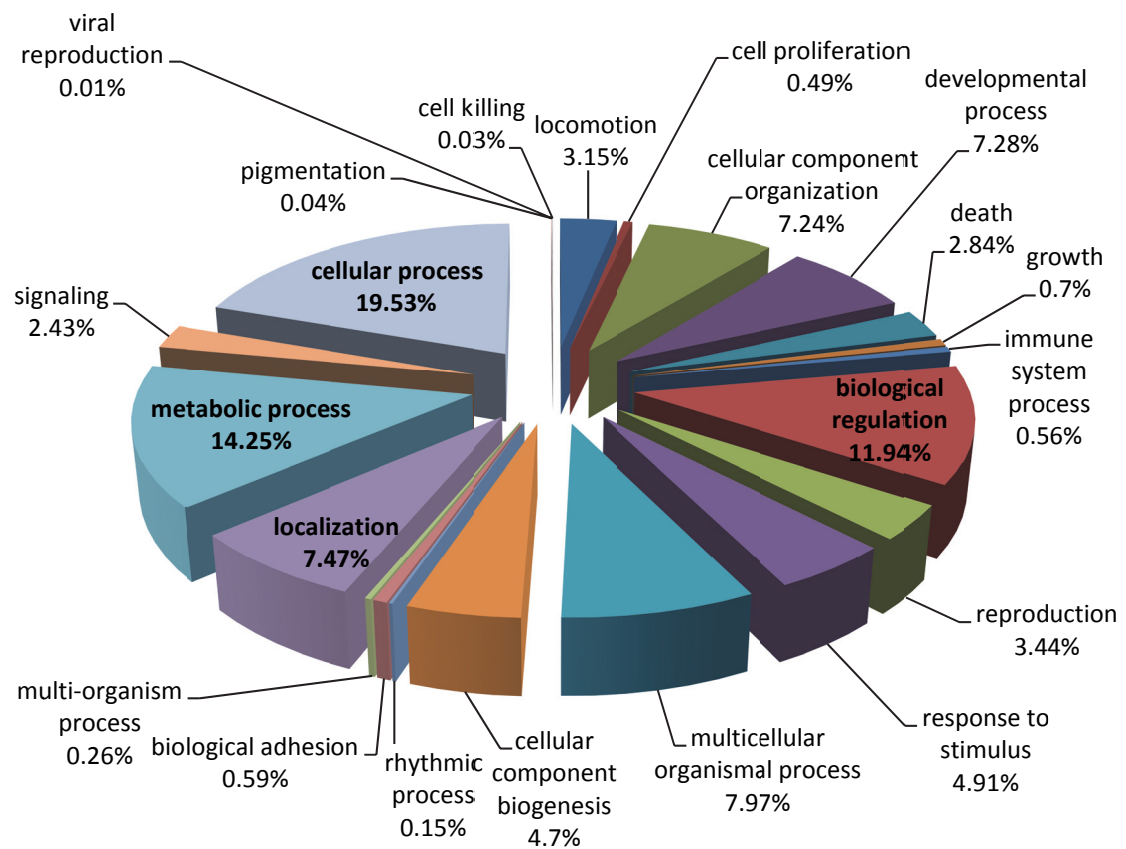

Supplement: Figure S2 — GO annotations for 3586 annotated F. merguiensis muscle/cuticle genes. Pie charts of a) molecular function (level 2), b) cellular components (level 3) and c) biological processes (level 2) of the annotated genes found to be expressed in the muscle/cuticle tissue. Blast2GO software was used for gene annotation. (PDF) [file pone.0056920.s002.pdf]

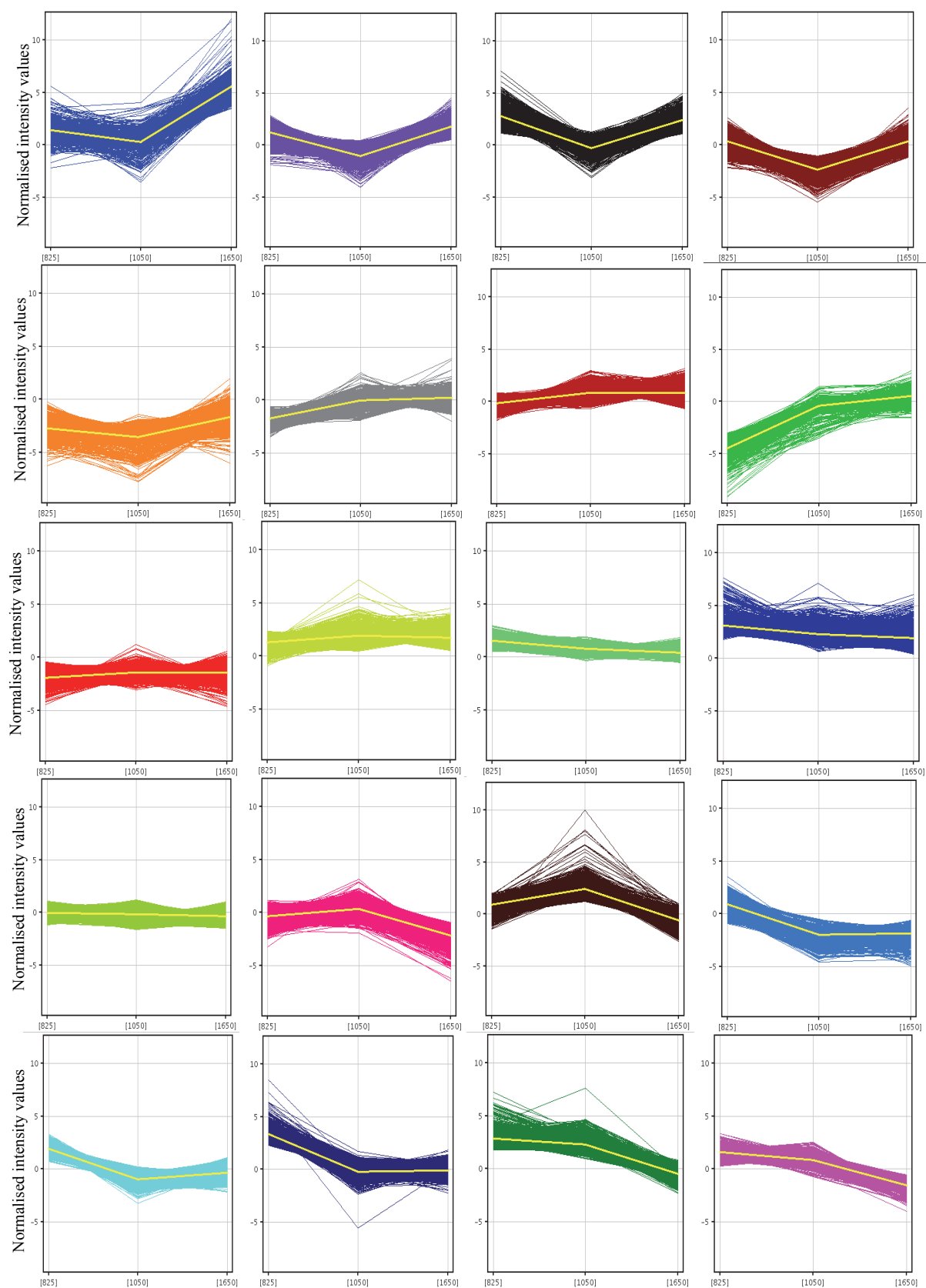

Supplement: Figure S3 — K-means (k = 20) cluster analysis performed across the albino groups. Vertical axis depicts normalised intensity values, horizontal axis shows the three different cRNA amounts (2×825 ng, 1050 ng and 1650 ng) used in the hybridisation reaction of the four albino sample groups, with the lowest concentration on the left and the highest on the right. Three of the albino groups were each comprised of cRNA from two pooled individuals, while the fourth albino group had three individuals contributing to the total cRNA amount (825 ng) for the hybridisation reaction. (PDF) [file pone.0056920.s003.pdf]

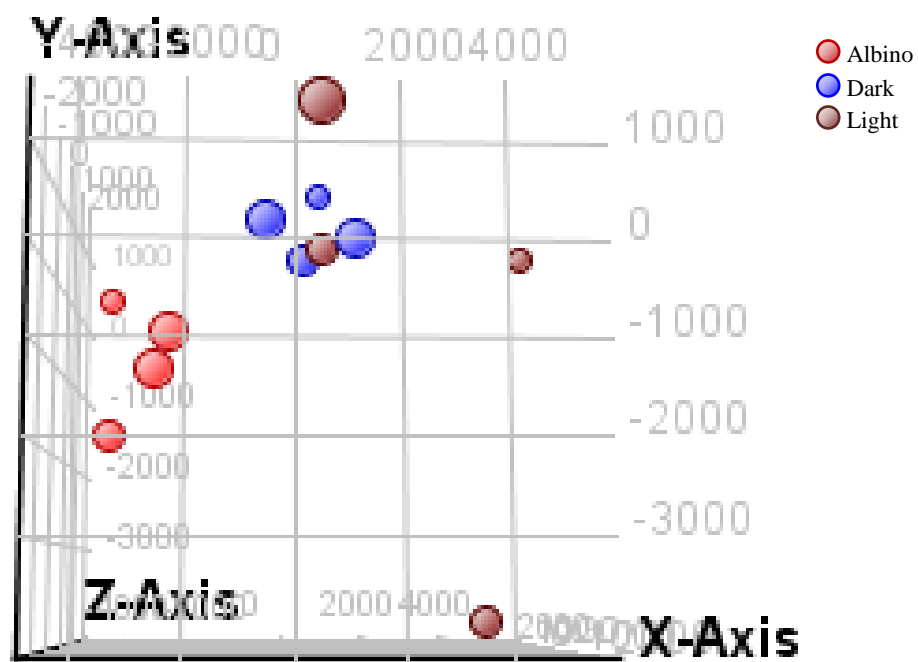

Supplement: Figure S4 — Principal component analysis (PCA) on albino, light and dark groups. The PCA plot shows the overall pattern of distribution of the four albino (red circles), four dark (blue circles) and light (brown circles) prawn colour groups. Component 1 is shown by the x-axis (44%), component 2 by the y-axis (22%) and component 3 by the z-axis (19%). Percentages in brackets are the variance explained by each axis. (PDF) [file pone.0056920.s004.pdf]

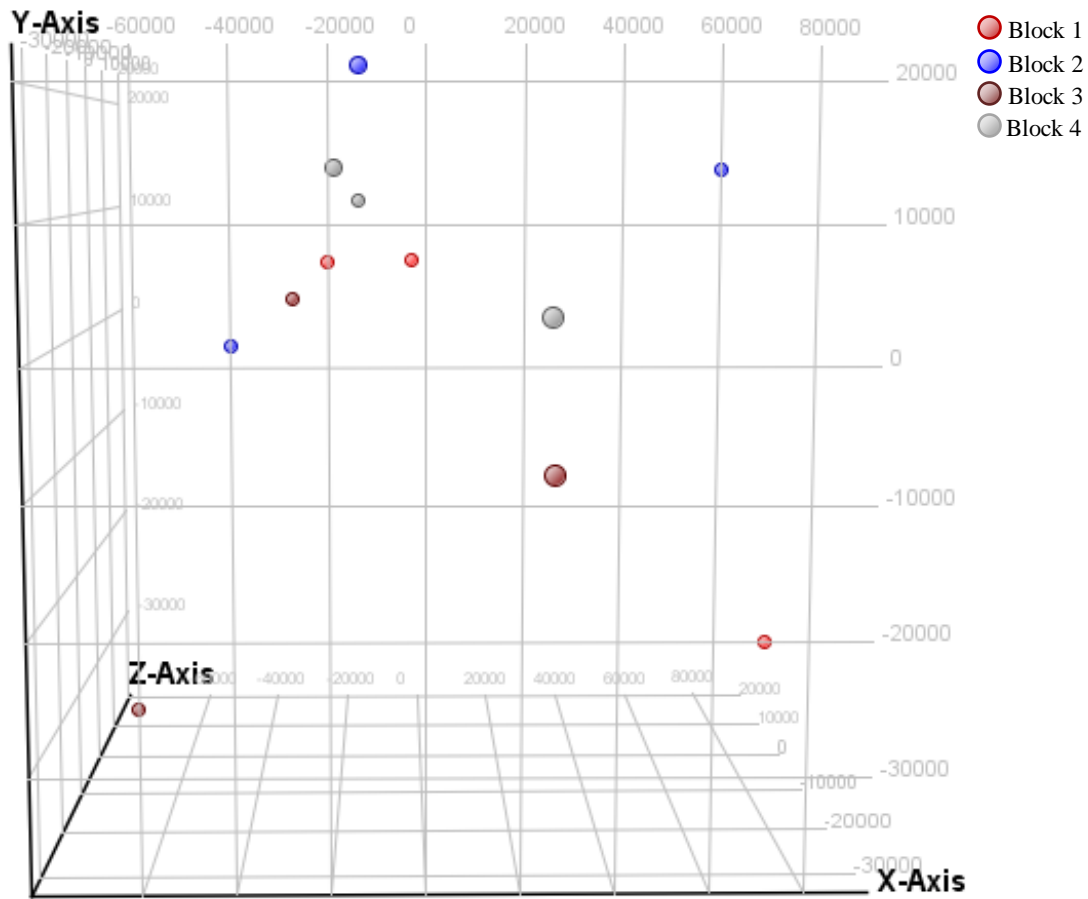

Supplement: Figure S5 — Principal component analysis (PCA) based on position of each block on the microarrays. The PCA plot shows the overall pattern of distribution of each of the hybridisation blocks on each of the three microarrays. PCA ordination was plotted with GeneSpring GX 12.5 (Agilent Technologies), depicting the relationships among samples on the basis of their respective locations on the microarrays: Block 1 (red circles, n = 3), Block 2 (blue circles, n = 3), Block 3 (brown circles, n = 3), and Block 4 (grey circles, n = 3). Component 1 is shown by the x-axis (explains 34% of the variation), component 2 by the y-axis (explains 12% of the variation) and component 3 by the z-axis (explains 10% of the variation). (PDF) [file pone.0056920.s005.pdf]
