# Supplementary material for: Molecular Characterisation of Colour Formation in the Prawn Fenneropenaeus merguiensis
Source: PLoS One. 2013 Feb 18;8(2):e56920. doi: 10.1371/journal.pone.0056920 (PMC3575496; doi:10.1371/journal.pone.0056920)
Supplement: Table S1 — Mutual expression profile of microarray probes differentially expressed across albino, light and dark prawns. Table shows probes that were found in the overlap areas of a Venn-diagram that graphically highlighted specific gene probes found in more than one of the single pair-wise comparisons (albino versus light, albino versus dark and light versus dark). The probes were found to be 2-fold or greater and statistically significantly (p<0.05) differentially expressed between albino and light and albino and dark F. merguiensis in at least two out of four probes. Results of the single pair-wise comparison of light versus dark, although not statistically significant, were included into the Venn-diagram and successive table to examine potential underlying biological trends. (PDF) [file pone.0056920.s006.pdf]

	Number of probes differentially expressed and common only between the below comparisons			
**Gene ID**	**Dark & Light and Albino & Light only**	**Dark & Light and Albino & Dark only**	**Albino & Dark and Albino & Light only**	**Common to all three comparisons**
arginine kinase/allergen Pen m	0	0	14	0
crustacyanin A	0	0	4	0
crustin	0	2	0	0
cytochrome c oxidase subunit I	2	0	0	0
male reproductive related protein	5	0	0	0
mitochondrion	2	2	0	0
myosin heavy chain	2	0	0	0
myosin light chain	2	0	0	0
myosin light chain or calmodulin	0	0	2	0
QM protein	2	0	2	0
ribosomal RNA	2	0	0	0
sarcoplasmic calcium-binding protein	2	0	17	3
some type of actin	0	4	44	12
tropomyosin	0	0	7	0
troponin I	18	0	3	0
uannotated	28	20	45	9
**Total**	**65**	**28**	**138**	**24**
