# Supplementary material for: Molecular Characterisation of Colour Formation in the Prawn Fenneropenaeus merguiensis
Source: PLoS One. 2013 Feb 18;8(2):e56920. doi: 10.1371/journal.pone.0056920 (PMC3575496; doi:10.1371/journal.pone.0056920)
Supplement: Table S2 — Individual crustacyanin subunit A and C copy numbers of the albino light and dark prawns. Mean individual copy numbers for each F. merguiensis prawn were determined with absolute qPCR, with each sample run in duplicate. (PDF) [file pone.0056920.s007.pdf]

ID #	Colour	Crustacyanin subunit A copy number	Crustacyanin subunit C copy number
173	Albino	1267.15	861.02
174	Albino	134.58	137.64
175	Albino	142.43	68.81
176	Albino	19.33	68.81
177	Albino	3345.61	530.31
180	Albino	19.33	68.81
181	Albino	3620.55	3724.27
182	Albino	19.33	68.81
183	Albino	19.33	68.81
1	Light	131984.84	230218.37
3	Light	18187.99	6480.19
6	Light	7341.53	5733.94
7	Light	92865.69	47290.65
14	Light	851892.31	1389450.38
15	Light	402159.52	386660.32
19	Light	94230.62	50019.29
20	Light	82934.33	54547.21
22	Light	57544.00	32276.43
24	Light	2702.33	667.43
25	Light	12148.26	5529.37
91	Light	6278.89	4704.82
11	Dark	9660.08	6572.52
30	Dark	108590.36	53990.80
86	Dark	205113.76	174745.35
87	Dark	66485.62	30461.60
88	Dark	62.34	68.81
90	Dark	2604.43	1048.08
93	Dark	9222.36	5464.88
95	Dark	49365.15	31459.62
97	Dark	6300.11	5117.33
101	Dark	5205.81	2627.47
104	Dark	68388.86	87326.61
109	Dark	5719.56	1815.07
